# GC Content Increased at CpG Flanking Positions of Fish Genes Compared with Sea Squirt Orthologs as a Mechanism for Reducing Impact of DNA Methylation

**DOI:** 10.1371/journal.pone.0003612

**Published:** 2008-11-13

**Authors:** Yong Wang, Frederick C. C. Leung

**Affiliations:** Department of Zoology and Genome Research Centre, The University of Hong Kong, Pokfulam, Hong Kong; Ecole Normale Supérieure de Lyon, France

## Abstract

**Background:**

Fractional DNA methylation in sea squirts evolved to global DNA methylation in fish. The impact of global DNA methylation is reflected by more CpG depletions and/or more A/T to G/C changes at CpG flanking positions due to context-dependent mutations of methylated CpG sites.

**Methods and Findings:**

In this report, we demonstrate that the sea squirt genes have undergone more CpG to TpG/CpA substitutions than the fish orthologs using homologous fragments from orthologous genes among *Ciona intestinalis*, *Ciona savignyi*, fugufish and zebrafish. To avoid premature transcription, the TGA sites derived from CGA were largely converted to TGG in sea squirt genes. By contrast, a significant increment of GC content at CpG flanking positions was shown in fish genes. The positively selected A/T to G/C substitutions, in combination with the CpG to TpG/CpA substitutions, are the sources of the extremely low CpG observed/expected ratios in vertebrates. The nonsynonymous substitutions caused by the GC content increase have resulted in frequent amino acid replacements in the directions that were not noticed previously.

**Conclusion:**

The increased GC content at CpG flanking positions can reduce CpG loss in fish genes and attenuate the impact of DNA methylation on CpG-containing codons, probably accounting for evolution towards vertebrates.

## Introduction

DNA methylation is typically pointed to cytosine methylation on CpG sites, although other types of DNA methylation were found in bacteria, fungi and insects [1], [2]. Principle biological function of DNA methylation in eukaryotes is regulation of gene expression. By means of methylating CpG sites within promoters, initiation of gene transcription is blocked and, in turn, abnormal demethylation on the promoters is responsible for cancers (see references in [3]). In view of molecular evolution, DNA methylation gives rise to CpG deficiency [4]–[6], because methylated CpG sites will easily mutate to TpG sites [7], [8]. Particularly in warm-blooded organisms, the number of CpG sites is significantly reduced because the deamination rate of methylated CpG is positively correlated to body temperature [4]. Notably, it was reported that the CpG mutations induced by DNA methylation are context-dependent; TA richness at the CpG flanking sites may accelerate the process 9, 10. Correspondingly, methylated CpG sites with flanking G/C (G or C) mutate at a lower rate, and therefore the context-dependence to some extent drives GC content increment at CpG flanking sites via mutational bias and/or positive selection [11].

DNA methylation level is gradually elevated at the boundary of invertebrates and vertebrates [12]. Fractional DNA methylation was discovered in sea urchin, sea squirt, amphioxus and lamprey; their genome was methylated at less than 50% level [12]. CpG methylation in these species, which is the same as that identified in vertebrates, was maintained by mammalian-like DNA methyltransferase 1 [13], [14]. Moreover, the DNA methylation in sea urchins has been suggested to be able to regulate embryonic development [15]. In sea squirts, reports demonstrated that repetitive elements could not be methylated, and DNA methylation was mainly found in transcribed regions [16], [17]. It was estimated that 80% of sea squirt genes are methylated and CpG deficiency has been observed at corresponding regions [17]. Fractional methylation in ascidians evolved to global methylation in fish, and over 90% of investigated fish genes were found to be methylated [12]. The global DNA methylation contributes the fish species with more precise regulation of gene expression to adapt to varying environmental factors. Fish genes can be silenced in specific tissues by methylating CpG islands in promoters due to the appearance of global DNA methylation, which might contribute to the variant densities and distributions of CpG islands among fish genomes [18]. The impact of DNA methylation on the promoters of the tissue-specific genes leads to lower CpG densities compared to housekeeping genes. A recent report shows that zebrafish tissue-specific and housekeeping genes can be distinguished by analyzing CpG densities of promoters as demonstrated in mammalian genes [19]. On the other hand, DNA methylation might have differentiated orthologous genes among the species at the invertebrate-vertebrate boundary due to the different levels of DNA methylation. Fish genes might undergo more CpG mutations and/or more A/T to G/C substitutions at CpG flanking positions than sea squirt genes, and raise numerous nonsynonymous substitutions and subsequent amino acid replacements. Therefore, the functional modification of gene products frequently occurred during the development from fractional to global DNA methylation, probably driving the evolution towards vertebrates.

To assess the impact of DNA methylation on genes, we studied orthologous genes of two sea squirt species and two fish species. The sea squirts are *Ciona intestinalis* (Ci) and *Ciona savignyi* (Cs); the fish include *Takifugu rubripes* (Tr; fugufish) and *Danio rerio* (Dr; zebrafish). Ci and Cs are invertebrate chordates that belong to the earliest branch in the chordate phylum (subphylum: Urochordata). A study reports that the CpG observed/expected (o/e) ratio of Ci genome is 0.85 and that of Tr and Dr genomes is about 0.5 [20]. Substitutions at CpG sites and the two flanking sites were collected from homologous sequences of the orthologous genes. We showed that Ci genes had more CpG substitutions than the orthologs of Cs, Tr and Dr. In contrast, fish genes were found to show obviously more T/A to G/C substitutions at CpG flanking positions, in such a way as to attenuate mutational pressure on CpG sites. This, in addition, was used to explain the extreme CpG deficiency in vertebrates. The nonsynonymous substitutions were then studied to evaluate their contribution to amino acid replacements.

## Results

### Increased GC1 and GC3 in fish genes

Without negative selection at the silent codon positions, the substitutions caused by CpG mutations at these positions were retained largely, and resulted in a decrease of GC3 (G+C content at the silent codon positions) in genes heavily methylated. We used homologous fragments obtained from orthologous genes among Ci, Cs, Tr and Dr to measure GC content at three codon positions. The sea squirt genes showed lower GC1, higher GC2 and lower GC3 (GC1 and GC2 denote G+C content at the first and second codon positions respectively) compared to fish orthologs (Table 1). GC3 difference was much more remarkable than GC1 and GC2 difference and the biggest GC3 difference (22%) was found in comparison of Ci*-*Tr. Adifference in the GC content was also observed between the sea squirts and between the fish species. Ci genes showed lower GC2 and GC3 than Cs genes; Tr genes showed higher GC3 than Dr genes. The high GC1 and GC3 in fish genes implicate that the sea squirt genes have accumulated more CpG mutations than the homologs in fish. Moreover, we expected to find more CpG mutations in Ci genes than in Cs genes, and in Dr genes than in Tr genes, in light of the GC content differences in Table 1.

**Table 1:** GC contents in three codon positions.

	Species pairs in comparison											
	Ci-Tr		Ci-Dr		Cs-Tr		Cs-Dr		Ci-Cs		Tr-Dr	
GC1	0.52	0.53	0.52	0.53	0.52	0.54	0.52	0.53	0.40	0.40	0.54	0.54
GC2	0.37	0.37	0.37	0.37	0.37	0.37	0.37	0.37	0.40	0.46	0.37	0.37
GC3	0.42	0.64	0.41	0.57	0.52	0.65	0.51	0.59	0.48	0.50	0.66	0.6

GC1, GC2 and GC3 denote G+C content at the first, second and third position respectively. Species pairs are the species in pairwise comparison in which homologous fragments were obtained for the calculation. The species include *C. intestinalis* (Ci), *C. savignyi* (Cs), *T. rubripes* (Tr) and *D. rerio* (Dr).

### More substitutions on CpG sites in sea squirt genes than in fish genes

To explain the increased GC content in fish genes, particularly GC3, we counted the number of CpG sites at different codon positions in the homologous fragments. The counting result of C1pG2, C2pG3 and C3pG4 (C3pG4 denotes the CpG located at the third position and the first position of the following codon) showed that fish genes contained more CpG sites at C3pG4 except in pairwise comparison between Cs and Dr, although the difference was not significant (Table 2). This partially explains the high GC3 in the fish genes and indicates a higher mutation rate of CpG sites in sea squirts. As to the amount of C1pG2 and C2pG3, the difference was small in comparison of Ci and the fish, because the biggest difference was less than 10% and sometimes the fish genes possessed more CpG sites. When Cs genes were compared to the fish genes, the difference was enlarged and Cs genes had more C1pG2 and C2pG3 sites than fish genes.

**Table 2:** Amount of CpG sites in different codon positions.

	Species pairs in comparison							
	Ci-Tr		Ci-Dr		Cs-Tr		Cs-Dr	
C1pG2	4683	4654	4463	4524	5905	5414	5504	5238
C2pG3	5188	5378	4846	4449	8517	6352	7688	5129
C3pG4	10543	14412	9537	10491	16846	17077	14870	12261

C1pG2 is used to denote CpG site on the first and second codon positions; C2pG3 is on the second and third codon positions; C3pG4 is on the third codon position and the first position of the next codon. The species pair and abbreviated species names were as shown in Table 1.

### Increased GC content at CpG flanking sites in fish genes

CpG mutation seems to be less frequent in fish genes, in which DNA methylation level is higher than in sea squirt genes. This is probably resulted from increment of GC content at CpG flanking positions.

To assess the significance of the GC3 increase before CGN codons in fish genes, we performed Fisher's exact test. In the test, T/A→G/C and G/C→T/A substitutions at the silent positions before conserved CGN codons were used to compare with those before non-CGN codons. The result showed that GC3 was significantly improved before the CGN codons in fish genes (see *P* values in Table 3). The same test was also performed on the third positions of CGN codons. GC3 increase in fish genes was found in all the comparisons, and only species pairs including Dr showed the significance (Table 4). It appears that T/A→G/C substitutions at 5′ flanking positions of conserved CpG sites are significantly more frequent in fish genes compared to sea squirt genes.

**Table 3:** Significant increase of GC content before CGN codons.

	Ci-Tr		Ci-Dr		Cs-Tr		Cs-Dr	
before CGN	CGN	others	CGN	others	CGN	others	CGN	others
A/T→G/C	472	21770	325	18021	429	20605	372	16879
G/C→A/T	108	7053	94	7860	177	10696	167	11518
*P* value	0.0012		0.0005		0.011		<10^−5^	

The datasets used are homologous fragments from the species pairs (see Table 1 for details). We counted A/T to G/C substitutions and G/C to A/T substitutions in fish genes. Particularly, the substitutions before conserved CGN codon and all the remaining silent codon positions were detected in the homologous fragments of the fish genes. Fisher's exact test was used to evaluate the difference in substitutions of A/T to G/C and G/C to A/T before the CGN codon.

**Table 4:** Significant increase of GC content at the third position of CGN codons.

	Ci-Tr		Ci-Dr		Cs-Tr		Cs-Dr	
within CGN	CGN	others	CGN	others	CGN	others	CGN	others
A/T→G/C	401	21734	347	17887	410	20458	366	16771
G/C→A/T	117	7000	100	7813	206	10614	198	11392
*P* value	0.35		0.0003		0.92		0.01	

Details are given in Table 3. The difference from Table 3 is that the silent positions within CGN codons were investigated, rather than before them.

### Strong negative selection on A nucleotides following TpG in sea squirt genes

The mutations of methylated CpG sites produced a large number of TpG sites in the sea squirt genes. We surprisingly found that A frequency was pretty low at the following position of TpG sites in sea squirt genes. In the test, the nucleotide frequencies at the TpG flanking positions of conserved NNT•GNN dicodons (the dicodon presents in both homologous fragments) were obtained using the homologous fragments between Ci and Cs. At the 5′ flanking position, the GC content was in accord with the overall GC2 in the two species, 38.9% and 45.9% respectively (Tables 1 and 5), whereas, at the 3′ flanking position, the GC content increased to 63%–68% and A frequency was dramatically low at only 4.9%. The 3′ A frequency was marginally lower than the 3′ T frequency and the 5′ A frequency (Table 5). The reason is perhaps the restriction against TGA in coding sequence in that an insertion or a dinucleotide deletion before the TGA will result in premature termination of the gene transcription. The result in Table 5 indicates that a large number of A nucleotides following the TGs have changed to G nucleotides due to perhaps strong positive selection. The proof is the remarkable difference between frequencies of G and C at the 3′ flanking position. The high GC content at this position also explains the high GC2 in the sea squirt genes (Table 1).

**Table 5:** Nucleotide frequency at flanking positions of dicodon NNT•GNN.

Species	5′ flanking position				3′ flanking position			
	A	T	G	C	A	T	G	C
Ci	0.301	0.31	0.20	0.189	**0.049**	0.323	**0.386**	0.242
Cs	0.267	0.274	0.228	0.231	**0.049**	0.272	**0.386**	0.293
Tr	0.349	0.271	0.167	0.213	0.212	0.376	0.195	0.216
Dr	0.352	0.269	0.166	0.213	0.212	0.376	0.195	0.216

Species names are described in Table 1. The results of Ci and Cs were obtained from homologous fragments between Ci and Cs, and those of Tr and Dr were obtained from homologous fragments between Tr and Dr. Conserved dicodon NNT•GNNs were located and nucleotide frequencies at the second positions of both codons were measured.

We also repeated the test using the dataset between the fish species. In contrast, the same result was not observed in fish (Table 5). Since a high percentage of the TpG sites in the sea squirt genes came from CpG mutations, the increment of 3′ G frequency was specially used by the sea squirt genes to cope with the impact of DNA methylation.

### Nonsynonymous and synonymous substitutions at NCGN sites

**Figure 1:**
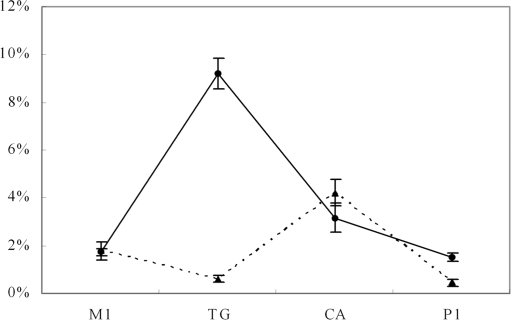
Percentage of CpG involved substitutions in pairwise comparisons. The test detected substitutions at NCGN sites in homologous fragments. The points on solid line represent percents of four types of substitutions to all that were detected on both homologous fragments, and those on dashed line show the results of nonsynonymous substitutions. The percentage is the average of the results using the homologous fragments from all pairwise comparisons among *C. intestinalis*, and *C. savignyi*, fugufish and zebrafish. The error bars are derived from standard deviation. Four types of substitutions on NCGN patterns are shown at x axis. The substitution type of M1 is referred to substitutions of T/A to G/C detected at −1 flanking position of CpG sites; P1 means the substitutions at +1 flanking position of CpG sites. TG and CA denote CpG to TpG and CpG to CpA respectively.

Using the homologous fragments, we counted synonymous and nonsynonymous substitutions at NCGN sites and non-CpG sites. The substitutions related to DNA methylation include CpG to TpG and CpA (TG and CA substitutions hereafter) and T/A to G/C at −1 and +1 flanking positions of CpG sites (M1 and P1 substitutions respectively hereafter). The percentage of these substitutions to all was calculated (Figure 1). Without specification of synonymous and nonsynonymous substitutions, TG substitutions were most frequently observed and accounted for 9.2% in average. CA substitution (3.2%) ranked the second, followed by M1 and P1 substitutions occupying 1.7% and 1.5% respectively. If restricted to nonsynonymous sites, the percentage of TG dramatically dropped to 0.6%, close to the lowest percentage of 0.44% for P1 substitution. CA substitution in this measure showed the highest percentage of 4.2%, followed by M1 substitution. The results suggest that a majority of TG and P1 substitutions are synonymous. Furthermore, the probability of nonsynonymous substitutions caused by TG is 11.7-fold lower than that caused by CA, and 15.3-fold lower than that caused by M1.

In the above test, the substitutions detected were a combination of those occurred within both homologous fragments from species X and Y (X and Y refer to any two of the four species). We next distinguished the substitutions in species X and Y, and found that the frequency of the four types of substitutions is different between the sea squirt and fish genes. TG and CA substitutions were more frequently observed in sea squirt genes; M1 and P1 substitutions occurred more frequently in fish genes. We counted the amounts of each substitution type in X and Y, and the ratio of the amounts between species X and Y was calculated for all substitutions and only nonsynonymous substitutions respectively (Table 6). Taking synonymous and nonsynonymous substitutions together, we found that Ci genes accumulated more TG and CA substitutions than all the other species, 1.23 times of those in Tr genes, 1.92 times of those in Dr genes, and 1.7 times of those in Cs genes. Differences in TG and CA substitutions were found between the fish as well. Tr genes had only about 60% of the substitutions detected in Dr genes. In contrast, the fish genes had more M1 and P1 substitutions, 2 to 5 times of those in the sea squirt genes (Table 6). Moreover, M1 and P1 substitutions in Cs genes were more than those in Ci genes; there are more in Tr genes than in Dr genes.

**Table 6:** Ratios from pairwise comparison of the amount of substitutions.

Substitution type	Species pairs in comparison (X-Y)					
	Ci-Tr	Ci-Dr	Cs-Tr	Cs-Dr	Ci-Cs	Tr-Dr
TG	1.92(1.32)	1.23(0.78)	1.14(0.93)	0.74(0.51)	1.72(1.36)	0.58(0.87)
CA	1.64(1.02)	1.33(1.11)	1.02(0.73)	0.79(0.74)	1.64(1.06)	0.66(0.81)
M1	0.27(0.59)	0.30(0.64)	0.47(0.73)	0.51(0.59)	0.61(0.78)	1.28(0.96)
P1	0.22(0.41)	0.25(0.21)	0.47(0.77)	0.52(0.64)	0.56(0.77)	1.39(1.08)

The ratios were calculated as the number of substitutions occurred in species X to that in species Y. The values in parentheses are specified to nonsynonymous substitutions. The abbreviations of species names are as indicated in Table 1 and substitution types have been described in Figure 1.

If only nonsynonymous substitutions were counted, the difference between species was generally narrowed compared to the results taking all substitutions into account (Table 6). The ratios for CA substitutions between Ci and the other three species decreased to around 1, as well as those for M1 and P1 substitutions between the fish, inferring that a large part of the difference in substitutions is ascribed to synonymous substitutions.

### Low CpG o/e ratios of fish genes are not totally a result of CpG mutations

**Figure 2:**
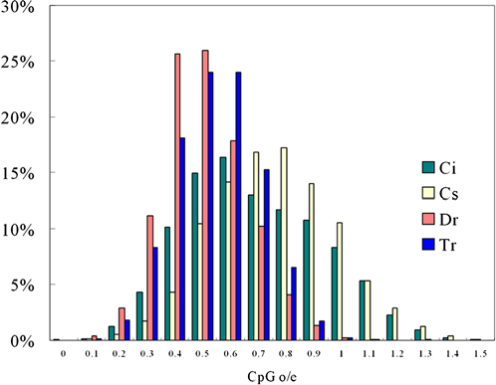
Percent of orthologous genes falling in different CpG o/e ranges. The abbreviated species names are as indicated in Table 1. The CpG o/e ratios at x axis represent o/e ranges in size of 0.1. The high CpG o/e ratios in sea squirt orthologous genes indicate that a large number of these genes (<40%) are not methylated.

In the above test, we found more TG and CA substitutions in sea squirt genes than in fish genes. However, the fish genes are more CpG deficient than the sea squirt genes in light of CpG o/e ratio (Figure 2). The mean CpG o/e ratios of the orthologous genes are 0.75±0.25 (standard deviation), 0.82±0.22, 0.59±0.15 and 0.55±0.15 for Ci, Cs, Tr and Dr respectively. According to Karlin's criteria for significance level of the values, CpG o/e value of Ci genes is significantly low; that of Cs genes is at normal level; those of the fish genes are very low [17]. We found that the inconsistency stems from using CpG o/e ratio to estimate CpG relative abundance in vertebrates. CpG o/e value is calculated as F_CpG_/(F_C_*F_G_), where F_CpG_ is frequency of CpG dinucleotide and F_C_ means frequency of C. When substitutions introduce C and G nucleotides into a DNA sequence with no chance of forming a CpG, CpG o/e value will decline. The same is for the process of increased GC content at CpG flanking positions, which results in declining CpG o/e ratio without loss of CpG sites. Therefore, the o/e ratios in the fish genes underestimate CpG frequency, and the CpG loss in vertebrates is not as significant as previously thought [6], [7].

**Figure 3:**
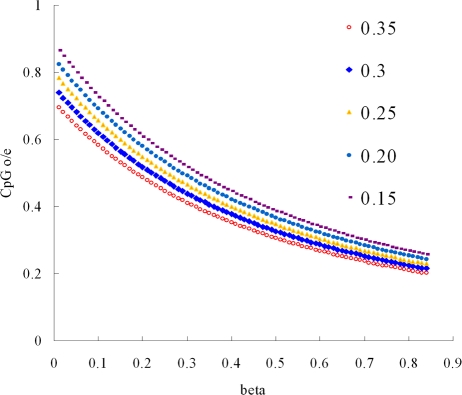
Substitutions of A/T for G/C at CpG flanking sites. CpG o/e denotes CpG observed/expected ratio. We computed the proportion (β) of T/A that changes to G/C at CpG flanking sites using an equation CpG o/e = (1−α)/((1−5α/24+β) ×(1−α/24+β)), where α is the proportion of mutated CpG sites in sea squirt genes. The prediction lines were drawn with five α values 0.15, 0.2, 0.25, 0.3 and 0.35.

We next developed a new equation for CpG o/e ratio, in which the percents of CpG loss and A/T to G/C changes at the CpG flanking positions were both incorporated. Assuming that CpG o/e ratio was 1 before DNA methylation and increased GC content at the CpG flanking positions could be neglected, we estimated the proportion (α) of mutated CpG sites using the mean o/e ratios of the sea squirt orthologous genes. The estimated α was 31% for Ci genes and 22% for Cs genes. We took the α of Cs genes as a reference for those of the fish genes and assumed that the two flanking positions of conserved CpG sites have equal opportunities in obtaining C or G (Figure 1). The proportion (β) of A/T to G/C changes at CpG flanking sites was estimated to be 22% and 17.5% for Tr and Dr genes respectively (see Methods). Using different α values, we plotted CpG o/e ratio against increased β (Figure 3). The figure may be used to estimate β for other vertebrates. For example, if CpG o/e ratio is 0.3 and α is 22% for a human gene, β is around 64%.

### Amino acid changes due to nonsynonymous substitutions

**Figure 4:**
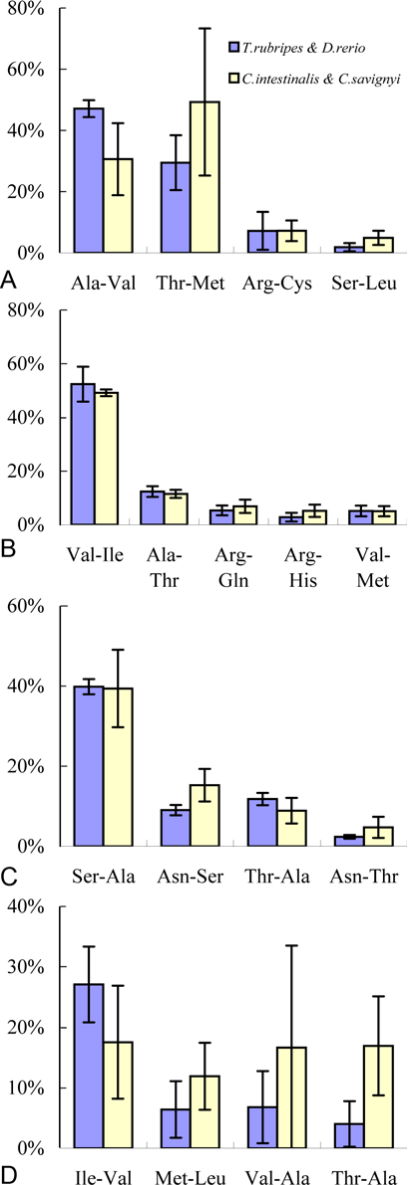
Most frequent amino acid changes due to CpG involved substitutions. The CpG involved substitutions point to TG, CA, M1 and P1 (see Figure 1 for details), and the results of these substitutions are shown in Figures 4A-D respectively. Amino acid changes are ranked on the basis of the percentages in all amino acid changes. We listed four to five of the highest ranked amino acid changes, and others were skipped due to low percentages. The blue columns show the results of amino acid changes occurred in fish genes, and the error bars derived from standard deviation were based on four pairwise comparisons between the fish genes and the sea squirt genes; the yellow columns represent the results from the sea squirt genes.

We then surveyed the nonsynonymous substitutions and the frequencies of amino acid replacements. Amino acid changes caused by TG substitutions in the fish genes were mostly found in Ala-Val (GCG-GTG) and Thr-Met (ACG-ATG) announcing 76% of all the changes, whereas more Thr-Met changes than Ala-Val changes were found in the sea squirt genes (Figure 3). The two types of amino acid changes were followed by Arg-Cys(CGY-TGY; Y = T/C) and Ser-Leu (TCG-TTG). Val-Ile (GTN-ATN except ATG) represented the major amino acid changes for CA substitutions (about 50%) and the type ranking the second was Ala-Thr (GCN-ACN) in about 17%. In nearly equal percentage of around 10%, the changes of Val-Met (GTG-ATG), Arg-Gln (CG[A/G]-CA[A/G]) and Arg-His (CG[T/C]-CA[T/C]) were resulted from CA substitutions (Figure 4). The result of M1 substitutions showed that Ser-Ala (TCG-GCG) represented the largest group of amino acid changes in 40%. They were followed by Asn-Ser (AAC-AGC), Thr-Ala (ACG-GCG) and Asn-Thr (AAC-ACC). Regarding P1 substitutions, the predominant change (27%) in fish genes was Ile-Val (ATN (except ATG) to GTN), and all the rest changes Met-Leu (ATG-CTG), Val-Ala (GTN-GCN) and Thr-Ala (ACN-GCN) occupied less than 10% of all. In contrast, the sea squirt genes showed no significant difference in the frequency of these amino acid changes, ranging from 10% to 20%. However, the occurrence of the amino acid changes was not stable among the species as indicated by the high standard deviations and there were other representative changes not shown (Figure 4). This was accounted for comparatively more directions of amino acid replacements in P1 substitutions. The high standard deviation was because of a small proportion of nonsynonymous substitutions in TG and P1 types (Figure 1).

We ranked amino acid changes induced by the substitutions of TG, CA, M1 and P1. The ranking results are similar for the fish and the sea squirt orthologs, although the frequencies of the four types of substitutions are different. This implies that the nonsynonymous substitutions have been strongly selected. The highly-ranked amino acid changes generally raise a weak conversion of amino acid properties such as acidity and polarity. The examples are the frequent changes between Val, Ile and Ala that show similar chemical and physical properties. The low frequency of the changes in other directions was probably caused by purifying selection.

We performed Fisher's exact test to determine which amino acid change showed a significant difference between the sea squirt and fish genes. All the potential changes caused by the substitutions (TG, CA, M1 and P1) were examined. The changes occurred at NCGN sites and non-CpG sites were detected for fish genes and sea squirt genes separately in order to constitute a 2×2 contingency table. All nonsynonymous substitutions showing a significant difference (*P*<0.05) in amino acid change between the fish and sea squirt orthologs were shown in Table 7. Most of the significant changes were attributable to M1 and CA substitutions. Six of them were among the highly ranked amino acid changes, including Ser-Ala, Ala-Thr, Val-Ile, Thr-Ala, Ala-Val, and Asn-Ser (Figure 4). Some amino acid changes, such as Asp-Asn and Ser-Pro, also show a significant difference although the frequency of the occurrence is low.

**Table 7:** Fisher's exact test for amino acid changes at NCGN sites and non-CpG sites.

species pair	Substitution	aa1→aa2		Non-CpG		NCGN		Significance
X-Y				X	Y	X	Y	level
Ci-Tr	M1	Ser	Ala	192	285	23	58	*
Ci-Tr	CA	Ala	Thr	132	78	35	40	*
Ci-Tr	CA	Asp	Asn	97	70	8	31	***
Ci-Dr	CA	Asp	Asn	119	78	6	15	*
Cs-Tr	CA	Val	Ile	765	680	156	231	***
Cs-Tr	M1	Ser	Pro	50	39	6	16	*
Cs-Tr	M1	Thr	Ala	102	85	14	26	*
Cs-Tr	TG	Ala	Val	45	35	9	19	*
Cs-Tr	M1	Asn	Ser	73	129	23	18	*
Cs-Tr	CA	Asp	Asn	96	73	27	46	**
Cs-Dr	CA	Val	Ile	844	697	144	214	***
Cs-Dr	CA	Asp	Asn	92	71	10	22	*

The abbreviated species names are as indicated in Table 1 and substitution types have been described in Figure 1. The results were obtained using homologous fragments from the species pair. Amino acid changes were collected at NCGN sites and other sites. Nonsynonymous substitutions responsible for the amino acid changes at NCGN sites include TG, CA, M1 and P1 substitutions. The numbers from species X and Y for Non-CpG and NCGN were used to constitute 2×2 contingency table. ^*^, *P*<0.05; ^**^, *P*<0.01; ^***^, *P*<0.001.

**Figure 5:**
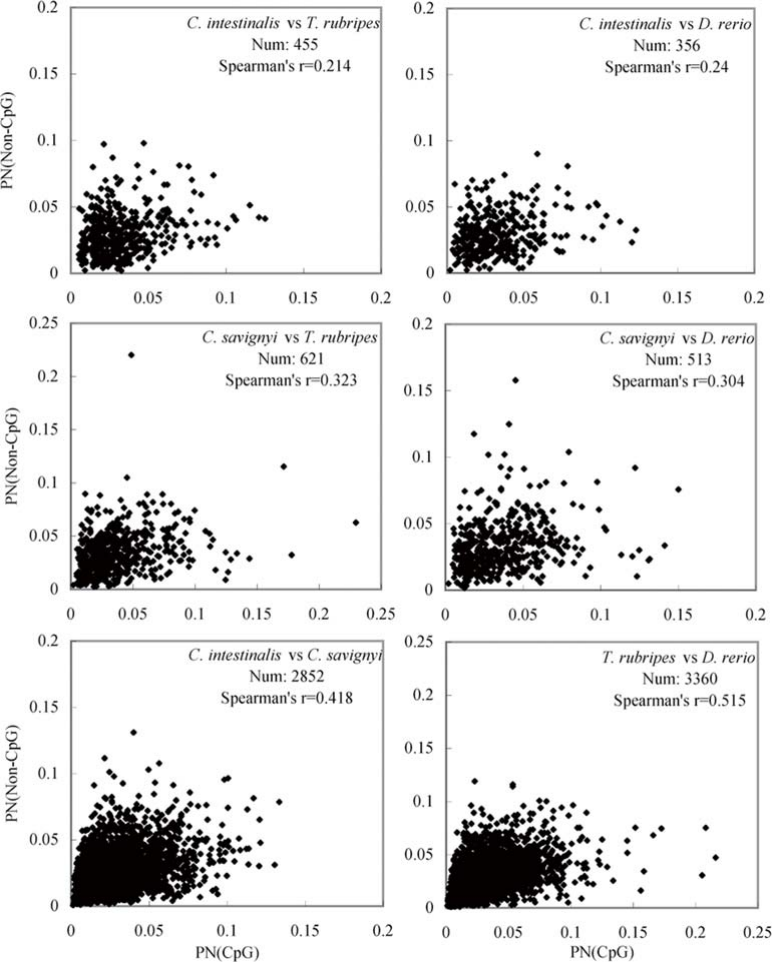
p_N_ plotting at NCGN sites and non-CpG sites. Homologous fragments, both containing more than 10 CpG sites, were used to calculate proportion of nonsynonymous difference (p_N_) at NCGN sites and the other sites separately. The Num in the figure means the number of homologous fragments in analyses. Nonsynonymous substitutions on NCGN sites include M1, TG, CA, and P1 (see Figure 1 for details about the substitution types). See Methods for calculation of p_N_. Spearman Rank Correlation test was used to evaluate the correlation between p_N_ values at NCGN sites and other sites (*P*<0.0001).

We observed a significant positive correlation between the nonsynonymous substitutions occurred at NCGN sites and those at non-CpG sites. Using the homologous fragments, the proportions of nonsynonymous differences (p_N_) were calculated for the orthologous genes. The plotting of the p_N_ values for NCGN sites and non-CpG sites was shown in Figure 5, in which the results of Spearman correlation tests were displayed. The results indicate that the correlation of the p_N_ values is significant (*P*<0.0001) in all the species pairs. In other words, the substitutions at NCGN sites are accompanied with those at the rest sites, or vice versa.

## Discussion

In this study, potential orthologous genes from sea squirts and fish were analyzed to assess the impact of DNA methylation on genes. Interestingly, sea squirt genes associated with fractional DNA methylation were found to accumulate more TG and CA substitutions than the fish genes with higher DNA methylation levels. We then discovered increased GC content at CpG flanking positions of the fish genes, which is supposed to protect the methylated CpG sites to some degree against spontaneous mutations. Our analyses convincingly support that the increased GC content is raised by context-dependent mutagenicity of methylated CpG sites [9], [10]. Nonetheless, we cannot totally preclude alternative explanations for this, such as a well-known hypothesis based on thermostability [21], [22]. However, recent studies produce results against a positive correlation between body temperature and GC content [23], [24]. In combination of all types of the substitutions occurred at CpGs and the flanking positions, the fish genes are found to show lower CpG o/e ratios than the sea squirt orthologs. Probably due to the above different substitutional patterns, the evolution of the orthologous genes has greatly diverged at the boundary of invertebrates and vertebrates.

The four types of substitutions related to DNA methylation could have affected protein products of the genes in three approaches. The first is protein component, i.e. loss and gain of amino acids. TG and CA substitutions result in loss of codons of NCG, CGN and GNN; M1 and P1 substitutions raise loss of [A/T]NN, N[A/T]C, [A/T]CG and G[A/T]N codons. Most frequently observed net gains because of TG and CA substitutions are codons of ATN, TTG and TGN; the gains because of M1 and P1 substitutions are GCN, CTN and GTN. Interestingly, some amino acid losses due to TG and CA substitutions are compensated by the gain from M1 and P1 substitutions, for example, Ala and Val. The process of increased GC content at CpG flanking sites in fish genes creates more M1 and P1 substitutions than sea squirt genes. Then, the frequent M1 and P1 substitutions in fish genes can help to balance amino acid component of gene products, rather than gain of some amino acids at cost of others as what have occurred in sea squirt genes.

The second approach is related to structural dynamics of gene products. Though the loss of the amino acids can be compensated elsewhere in fish genes and the overall component of amino acids probably does not change, the positions of the relevant amino acids are shuffled frequently. Hence, the establishment of DNA methylation facilitates structural modification of gene products. This effect is not significant in sea squirts because the loss of some amino acids caused by TG and CA substitutions cannot be compensated via other frequent amino acid replacements. Corresponding to the four types of substitutions occurred at NCGN sites, a considerable number of substitutions at non-CpG sites have probably been positively selected for maintenance of proper and stable tertiary structures of proteins. In this study, we have shown that the proportion of nonsynonymous substitutions occurred at NCGN sites is significantly correlated with that at non-CpG sites (Figure 5). Therefore, the nonsynonymous substitutions at non-CpG sites are also the sources of amino acid changes used to balance the component of the protein sequences.

Finally, length of gene products may be strongly affected as well. In case that the last codon before stop codon is NCG, the stop codon will probably be converted to GAA, GAG or GGA in fish genes, and thus the reading-frame is extended. The reading-frame may also be shortened if one of CGA codons becomes a TGA codon. Local GC content is a determinant factor because 1) a gene ending with a high GC content tends to extend its reading-frame; 2) a gene with low GC content regions tends to shorten its reading-frame. Therefore, GC content of vertebrate genes gradually declines as shown by previous reports [25], [26]. Moreover, we found that starting codon ATG may be generated and depleted by the substitutions, raising a probability of moving the starting point of coding regions to elsewhere.

In this study, we clearly show that sea squirt and fish orthologs much differ in the substitutional patterns under the impact of DNA methylation. The question is what makes the difference. On the basis of primary work, we propose that it is probably due to the presence of a critical gene encoding MDB4 (methylated DNA-binding domain 4) protein for repairing mutations on methylated CpG sites in fish species. Mammalian MDB4 was found to be able to efficiently correct the mutations caused by deamination of methylated CpG [27]–[29]. Till now, the presence of the MBD4 gene has not been confirmed in the fish species, but zebrafish does have a few MBD genes such as Mecp2, MBD2, MBD3a, and hypothetical proteins with the methylated DNA-binding domain (through BLAST search in the NCBI). Given the repair work by the MBD4-like protein, the mutation rate of CpG sites will be fundamentally decreased, providing sufficient evolutionary time for positive selection of random A/T to G/C mutations at CpG flanking positions. In sea squirts, the MBD4 gene probably has not evolved at the stage of fractional DNA methylation in their genomes. The BLAST result using human MBD4 protein did not show any proteins with the methylated DNA-binding domain in Ci and Cs. A report shows that inactivity of MBD4 gene in mice will result in 2- to 3-fold increase of C to T mutations [21] [30]. Therefore, lacking the MBD4 gene in sea squirts could have resulted in a large number of CpG mutations in a short evolutionary history, and then the new TpG sites in sea squirt genes drive a strong negative selection against the following A nucleotides due to the potential to form a stop codon. The much lower A frequency following TpG sites compared to that in fish genes is perhaps due to more frequent insertions or deletions that shift reading-frames of the sea squirt genes.

Our study also revisits the issue of the extreme CpG deficiency in vertebrates and clarifies that a low CpG o/e ratio does not equate to the degree of CpG loss. Genomic variation of GC content and CpG dinucleotide is an issue hotly debated. Previous studies have proposed many mechanisms for the observations in prokaryotic and eukaryotic genomes [7], [8], [31]–[33]. No individual hypotheses can successfully explain the phenomenon in all species involved. DNA methylation is a widely accepted hypothesis at present. In this study, we show that a low CpG o/e ratio in fish orthologs is partially caused by increased GC content at CpG flanking positions, in light of context-dependent mutations of methylated CpG sites. To what a degree that the increased GC content contributes to the low ratio depends on local GC content. In high-GC content regions, a low CpG o/e ratio is mainly stemmed from accumulated G/C at CpG flanking positions; in low-GC content regions, CpG mutational rate is high and CpG o/e ratio reflects the degree of CpG depletions to a higher extent. Thus, our finding supports the causal role of DNA methylation in CpG deficiency, but advocates caution in usage of CpG o/e ratios for evaluation of CpG deficiency particularly in vertebrates.

Our finding is probably useful to explain CpG deficiency in genomes of bacteria, viruses and mitochondria [34]–[36]. Because CpG depletions caused by the DNA structural constraints are context-dependent as well [31], increased GC content at CpG flanking positions in bacterial and mitochondrial genomes are also highly expected. In mammalian genomes, the G/C clustering process around CpG sites seems to be causal to the formation of CpG islands and GC-rich isochores [37], [38].

## Materials and Methods

### Collection of orthologous genes

Using Ensembl 42 Homology Database in BIOMART (http://www.biomart.org/), we obtained orthologue tables containing pairwise orthologous genes for the following species pairs: Ci (JGI2) and Cs (CSAV2), Ci and Dr (ZFISH6), Ci and Tr (FUGU4). To remove redundancy, we only kept the first orthologue pair if a gene had multiple orthologues in another species. We compact the tables into one by matching the Ci IDs between the tables. Thus, we had 5968 orthologous genes from the four species. The protein and coding DNA sequences (CDSs) of the orthologous genes were downloaded from the EMBL (http://www.ensembl.org/).

### Extraction of homologous fragments

We executed pairwise alignments on the orthologous genes from six species pairs that include all combinations of the four species: Ci, Cs, Tr and Dr. We first extracted homologous fragments from CDSs of the orthologous genes. The alignment starts from finding an identical sequence seed of 5 bp on both sequences. Homologous fragments were obtained from extension of the seed at both ends. The extension terminated while two continuous mismatches were found. Homologous fragments longer than 30 bp were taken into a dataset and then translated into three versions of protein sequences using different reading-frames. Correct translations of the homologous fragments were identified by aligning them on protein sequences of the genes in comparison. If the translation was confirmed for both species, the DNA fragments within the correct reading-frame and the translated peptides were collected. The alignment could be resumed at a new site until the searching reached the end of CDSs. Therefore, we sometimes might collect more than one homologous fragment in a single sequence pair. In analyses of GC content and CpG amount, we concatenated the homologous fragments first.

### Detection of excessive amino acid changes caused by DNA methylation

We recorded all the nonsynonymous substitutions between the homologous fragments. The substitutions involved in DNA methylation were referred to those on CpG sites and the flanking positions (NCGN). Fisher's exact test was performed on the data of mutual amino acid (AA) changes (AA1 to AA2 and AA2 to AA1) caused by the nonsynonymous substitutions at the NCGN sites and the remains. The AA changes caused by M1, TG, CA, and P1 substitutions were used to calculate the significant difference separately.

### Calculation of p_N_ at NCGN sites and other sites

Proportions of nonsynonymous differences (p_N_) were measured on NCGN sites and the remaining sites. The substitutions on NCGN sites were referred to M1, TG, CA, and P1 substitutions. We selected the homologous fragments that both contain at least 10 CpG sites for the test. The p_N_ was calculated as Nd/N, where Nd was the number of nonsynonymous differences between the homologous fragments and N was the average number of nonsynonymous sites for both of the fragments. Nd and N were obtained as described elsewhere [39]. In this study, we measured p_N_ for NCGN sites and the others separately in order to assess significance of the correlation of the p_N_ values.

### CpG o/e value and estimate of substitutions at CpG and flanking sites

CpG o/e value was first measured using formula F_CpG_/(F_C_×F_G_), where F_CpG_ denotes frequency of CpG and F_C_ denotes the frequency of C [34]. Assuming that CpG o/e ratio was 1 for the sea squirt genes before DNA methylation, and therefore the proportion (α) of CpG depletions caused by DNA methylation could be estimated using a new definition of the o/e ratio. Given a DNA sequence in size of L, GC content = ρ and G% = C%,
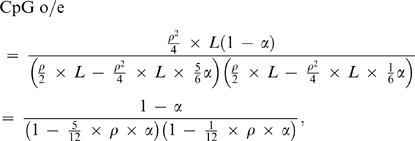
where substitutions on C were set to be five-fold of those on G in CpG sites (unpublished result). When GC content = 0.5, o/e = (1−α)/((1−5α/24)×(1−α/24)). We then computed proportion (β) of substitutions of A/T to G/C at CpG flanking sites in the fish genes. The equation is o/e = (1−α)/((1−5α/24+β)×(1−α/24+β)), where α is the proportion of CpG depletions in the sea squirt genes.
